# Progeny fitness determines the performance of the parasitoid *Therophilus javanus*, a prospective biocontrol agent against the legume pod borer

**DOI:** 10.1038/s41598-021-88644-3

**Published:** 2021-04-26

**Authors:** Djibril Aboubakar Souna, Aimé Hippolyte Bokonon-Ganta, Marc Ravallec, Mesmin Alizannon, Ramasamy Srinivasan, Barry Robert Pittendrigh, Anne-Nathalie Volkoff, Manuele Tamò

**Affiliations:** 1 International Institute of Tropical Agriculture, Benin Research Station (IITA-Benin), 08 BP 0932 Tri Postal, Cotonou, Benin; 2 Department of Crop Production, Faculty of Agronomic Sciences (FSA), University of Abomey-Calavi (UAC), 03 BP 2819 Cotonou, Benin; 3 UMR DGIMI University of Montpellier, INRAE, Place Eugène Bataillon, 34 095 Montpellier, France; 4 World Vegetable Center (WorldVeg), Shanhua, Tainan 74151 Taiwan; 5 Department of Entomology, Michigan State University (MSU), East Lansing, USA

**Keywords:** Agroecology, Behavioural ecology, Ecophysiology

## Abstract

*Therophilus javanus* (Bhat & Gupta) is an exotic larval endoparasitoid newly imported from Asia into Africa as a classical biological control agent against the pod borer *Maruca vitrata* (Fabricius). The parasitoid preference for the five larval instars of *M. vitrata* and their influence on progeny sex ratio were assessed together with the impact of larval host age at the time of oviposition on development time, mother longevity and offspring production. In a choice situation, female parasitoids preferred to oviposit in the first three larval instars. The development of immature stages of the parasitoid was observed inside three-day-old hosts, whereby the first two larval instars of *T. javanus* completed their development as endoparasites and the third larval instar as ectoparasite. The development time was faster when first larval instars (two- and three-day-old) of the host caterpillars were parasitized compared to second larval instar (four-day-old). The highest proportion of daughters (0.51) was observed when females were provided with four-day-old hosts. The lowest intrinsic rate of increase (*r*) (0.21 ± 0.01), the lowest rate of increase (λ) (1.23 ± 0.01), and the lowest net reproductive rate (*Ro*) (35.93 ± 6.51) were recorded on four-day-old hosts. These results are discussed in the light of optimizing mass rearing and release strategies.

## Introduction

*Therophilus javanus* (Bhat & Gupta) (Hymenoptera: Braconidae) is a solitary endoparasitoid attacking larval stages of the cowpea pod borer *Maruca vitrata* (Fabricius) (Lepidoptera: Crambidae)^1^. This parasitoid was recently imported from Taiwan into Benin as a candidate biological control agent against the cowpea pod borer in West Africa^2^. Knowledge about life history traits—i.e., the main biological parameters that can affect the reproductive function and survivorship of an organism—is fundamental for understanding how organisms adapt to their environment. In the case of a parasitoid, this knowledge is a prerequisite prior to its introduction in a novel environment as a biological control agent^3^. Already, the biological potential of this newly introduced natural enemy has been investigated with regard to reproductive biology^4^ and the ability of this natural enemy to localize a plant-feeding *M. vitrata* caterpillar^5^. However, life traits estimation could provide additional and critical information on quantitative and ecological function of natural enemies^6^, and population growth can be estimated by assessing fertility life table parameters^7^. These parameters, when known for both a pest and its natural enemy, may also help to plan the outcome of their interaction^8^. Although *T. javanus* has been known in Asia for a long time^2^, there has been no attempt to date to the authors’ knowledge to study *T. javanus* life history traits in general.

Different factors can shape parasitoid life history trait: host quality (size, age, or stage)^9^, parasitoid feeding strategies during larval stage^10,11^ and adult feeding behavior^12^. Because *T. javanus* parasitizes a single host, its suitability and the amount of nutritious resources it provides, will be a determinant for parasitoid survival and development. Thus, some parasitoids should maximize food consumption during larval stage and ultimately favor adult fitness^13^. Along this line, two different larval feeding strategies have been reported in koinobiont endoparasitoids^14^. Some complete part of their larval stages inside the host body, but after egression from the host at a late larval stage destructively consume all (or most) of the host tissues before formation of cocoon (“tissue feeders”)^10^. Others complete their larval stages inside the host by feeding nondestructively on the host hemolymph or fat body and then egress from the host to form a cocoon on, or close, to the host, which may remain active and live temporally before dying (“hemolymph feeders”)^15^. Overall, hemolymph-feeder parasitoids seem to be more advantaged because they can exploit a wide range of host stages and can use the dying host as a bodyguard against predators and hyperparasitoids after egression^16^. However, in tissue feeder parasitoids, feeding after egression can also enhance adult fitness^10^. Consequently, for both tissue feeders and hemolymph feeders, the quality of the host parasitized by the mother parasitoid can affect progeny fitness, including immature survival, development, adult longevity, and fecundity^17,18^.

Host quality at the time of oviposition and parasitoid larval feeding strategies are known to significantly influence adult fitness^19,20^. The objectives of the present study were to determine the larval feeding behavior in *T. javanus*, investigate the impact of the host age on total development time, mother female longevity and offspring production, sex-ratio of the progeny, and estimate the life table parameters under laboratory conditions.

## Results

### Brief description of Therophilus javanus

**Figure 1:**
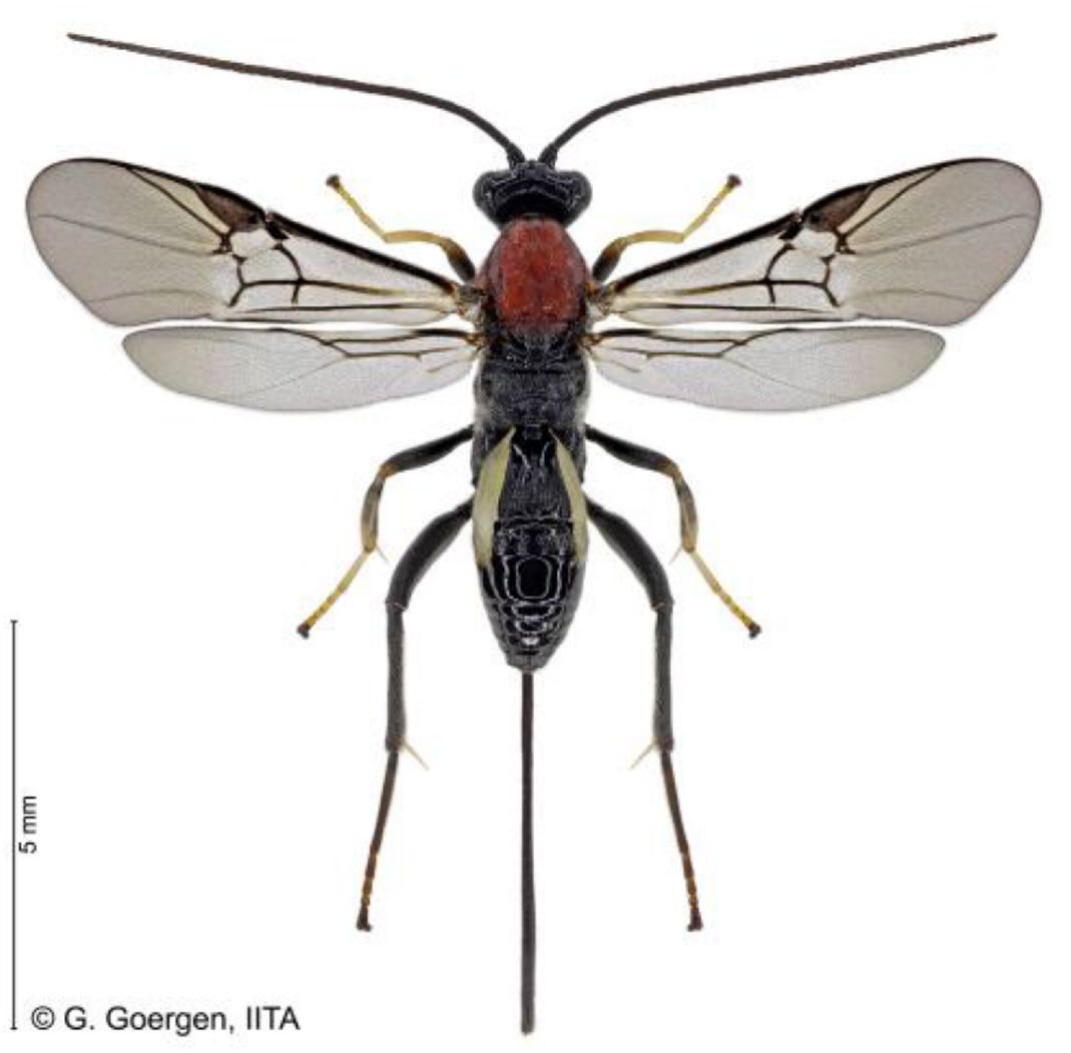
*Therophilus javanus* (Bhat & Gupta), female, Goergen Georg/IITA Benin. Scale bar = 5 mm.

*Therophilus javanus* is a braconid parasitoid of Agathidinae subfamily with a long ovipositor sheath (Fig. 1). The mesonotum of the parasitoid is dark orange, the metasoma is black, while the first to third metasomal sternites are whitish (see^21^ for more details).

### Host stage preference and progeny sex ratio

Host stage preference and the sex ratio—i.e. the ratio of males to females in the progeny — of the parasitoid were significantly affected by the larval instar of *M. vitrata* caterpillar (Table 1). Parasitization attacks and subsequent oviposition occurred more frequently on first and second larval stages, and less often on third larval instars (GLM: χ2 = 132.3, df = 4, *p* < 0.001). The female did not oviposit on the fourth and the fifth larval instars during the observation. The sex ratio was significantly influenced by the host larval instar chosen by the female at the time of oviposition (χ2 = 7.0343, df = 2, *p* = 0.03). More males emerged from *M. vitrata* caterpillars parasitized at the first larval instar (χ2 = 5.7647, df = 1, *p* = 0.02). However, there were no significant difference between the number of males and females when the host was parasitized at the second or the third larval instar (χ2 = 0.80645, df = 1, *p* = 0.4) and (χ2 = 0.72727, df = 1, *p* = 0.4), respectively.

**Table 1:** Percentage of successful oviposition (mean ± SE) and sex ratio of *T. javanus* in choice situation among the five instars of *M. vitrata* caterpillars.

Host larval instar	Oviposition	Sex ratio
First	0.40 ± 0.10a	2.4a
Second	0.40 ± 0.14a	0.72b
Third	0.20 ± 0.08b	0.69b
Fourth	0	–
Fifth	0	–

Means ± SE of oviposition followed by different letters in the same column are significantly different among treatments (GLM and Tukey’s HSD test, *p* < 0.05). Sex ratio followed by different letters in the same column are significantly different among treatments (chi-square test followed by ‘pairwise nominal independence’).

### Larval feeding behavior and developmental time of T. javanus immature stages

At 26 ± 1.1 °C, *T. javanus* egg development lasted an average 1.94 ± 0.16 days (n = 45). Larval development included three larval instars. The duration of the first larval instar was 3.04 ± 0.21 days (n = 137). The second and third larval instars lasted 2.04 ± 0.21 days (n = 92) and 1.84 ± 0.37 days (n = 83), respectively.

### Endoparasitic feeding

**Figure 2:**
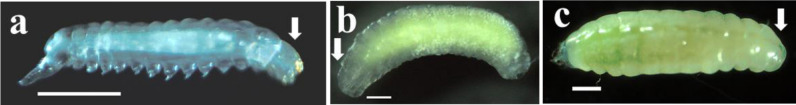
Larva of *T. javanus*, (arrow indicates the head): (**a**) first instar, (**b**) second instar, (**c**) third instar. Scale bar = 5 mm.

First instar larvae were translucent, polypodeiform, with thirteen body segments and a distinct sclerotized head capsule harboring a pair of prominent mandibles (Fig. 2a). Each body segment except the last one presented two pairs of ventral processes of uniform size. The last segment was prolonged by a small, ventrally curved tail. The gut was visible, and content changed in color from translucent in early first instar to white or green in the late first instar of *T. javanus* reared on *M. vitrata*. The second instar was of hymenopteriform type (Fig. 2b). The head was hemispherical with no apparent mandibles. At this stage, there were no more visible paired ventral processes and the ventrally curved tail was lost. The larva was opaque white, and the gut was green. The early third instar was also of hymenopteriform type. The body was opaque white, but the gut was green and contained white granules (Fig. 2c).

### Ectoparasitic feeding

**Figure 3:**
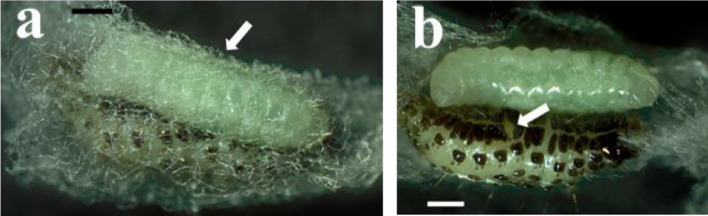
Third instar *T. javanus* larva egressed from *M. vitrata* caterpillar. (**a**) *T. javanus* larva, white colored, feeding on *M. vitrata* caterpillar within the *M. vitrata* cocoon indicated by an arrow; (**b**) arrow indicating the egression hole on *M. vitrata* caterpillar (picture taken after host cocoon has been removed). Scale bar = 5 mm.

About eight days after parasitism, the third instar larva egressed from the host to become ectoparasitic (Fig. 3a). The body color remained opaque white and the gut turned from green to yellow during this last part of the larval development. At this stage, the larval body was cylindrical, narrowed at both ends, and the anterior end was curved. Egressed larvae fed on the host larva by sucking all the host contents through the egression hole (Fig. 3b).

### Cocoon and pupa formation

**Figure 4:**
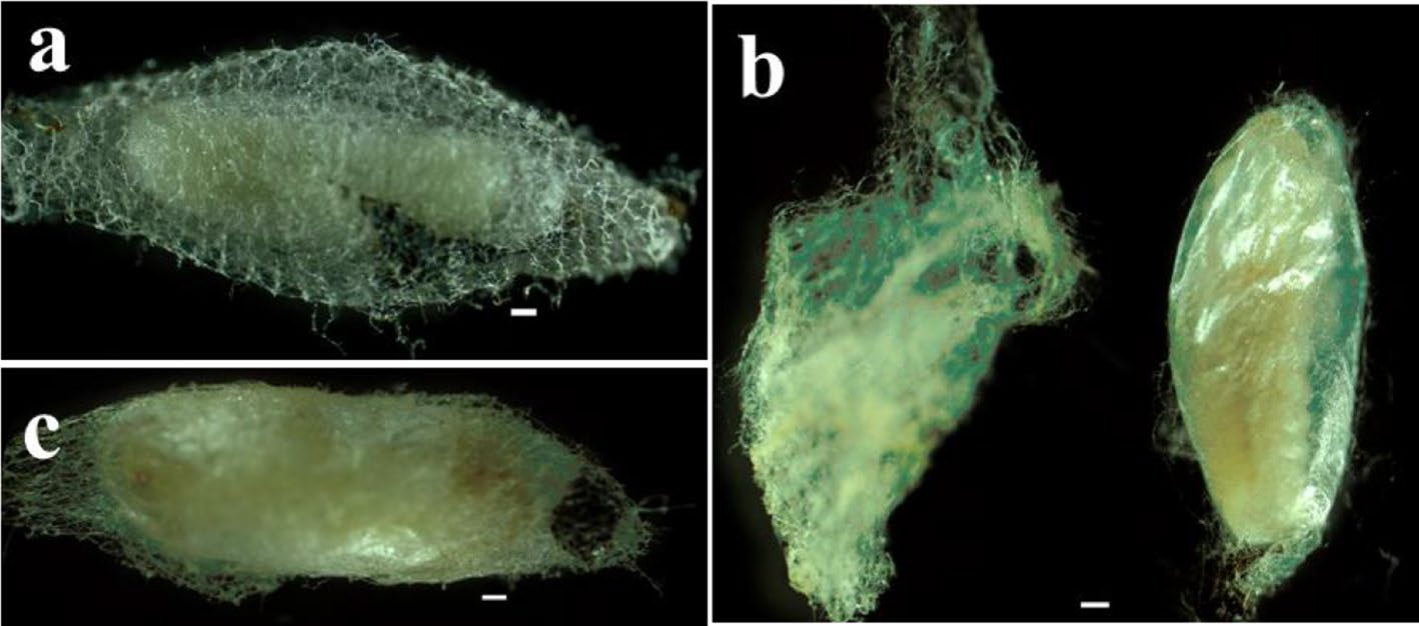
Morphology of *T. javanus* cocoon. (**a**) *T. javanus* spins the cocoon within the silken cocoon of *M. vitrata*; (**b**) view of the newly formed *T. javanus* cocoon (right) dissected from the cocoon of *M. vitrata* (left); (**c**) view of the fully formed cocoon of *T. javanus*. Scale bar = 5 mm.

**Figure 5:**
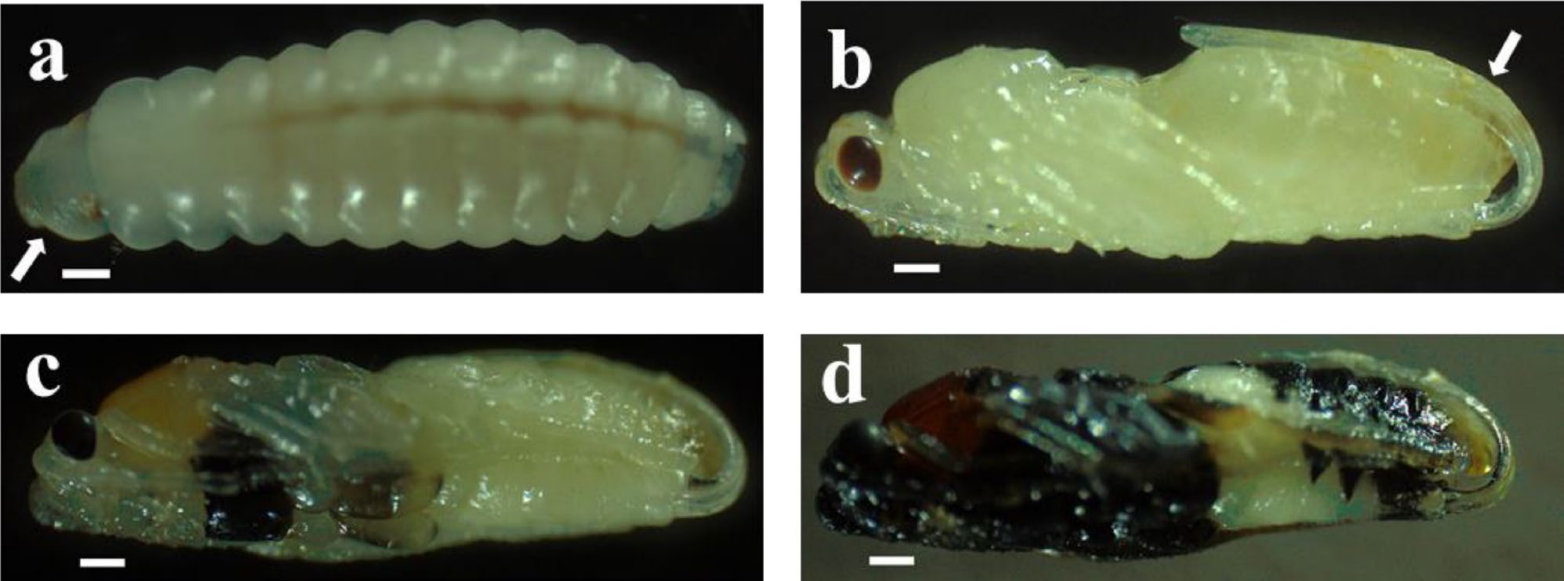
Progressive body changes and pigmentation during pupation of female *T. javanus*. (**a**) pre-pupa, with arrow indicating the head; (**b**) hyaline pupa, with arrow indicating the ovipositor; c-d) progressive body pigmentation during pupation. Scale bar = 5 mm.

After the egressed larva emptied the host contents, the late third instar spun a cocoon (Fig. 4) before entering in pre-pupal stage. The pre-pupa instar displayed a dirty-white color and was identified when the head, thorax, and abdomen of the future adult started to be distinguishable (Fig. 5a); its duration was 1.11 ± 0.32 days (n = 50). The color of the newly formed pupa turned into ivory-white within a cocoon after expelling the brown meconium contained in the gut (Fig. 5b). During pupation, pigmentation was progressive and started from the head region, then reached the thorax, the abdomen, the legs, the antenna and, finally, the ovipositor (for the females) (Fig. 5c, d). Total duration of pupal stage was 5.65 ± 0.82 days (n = 41).

### Impact of host age on adult parasitoid development time

The total development time (from egg to adult) was found to be significantly influenced by the age of the host at the time of oviposition (GLM: χ2 = 0.32489, df = 2, *p* < 0.001). *Therophilus javanus* development time was faster when two- and three-day-old (first larval instars) *M. vitrata* caterpillars were parasitized [15.35 ± 0.78 days (*n* = *85*) and 15.43 ± 0.91 days (*n* = *74*), respectively] compared to four-day-old (second larval instar) *M. vitrata* caterpillars (16.73 ± 0.82 days; *n* = *64*).

### Impact of host age on mother parasitoid longevity

**Figure 6:**
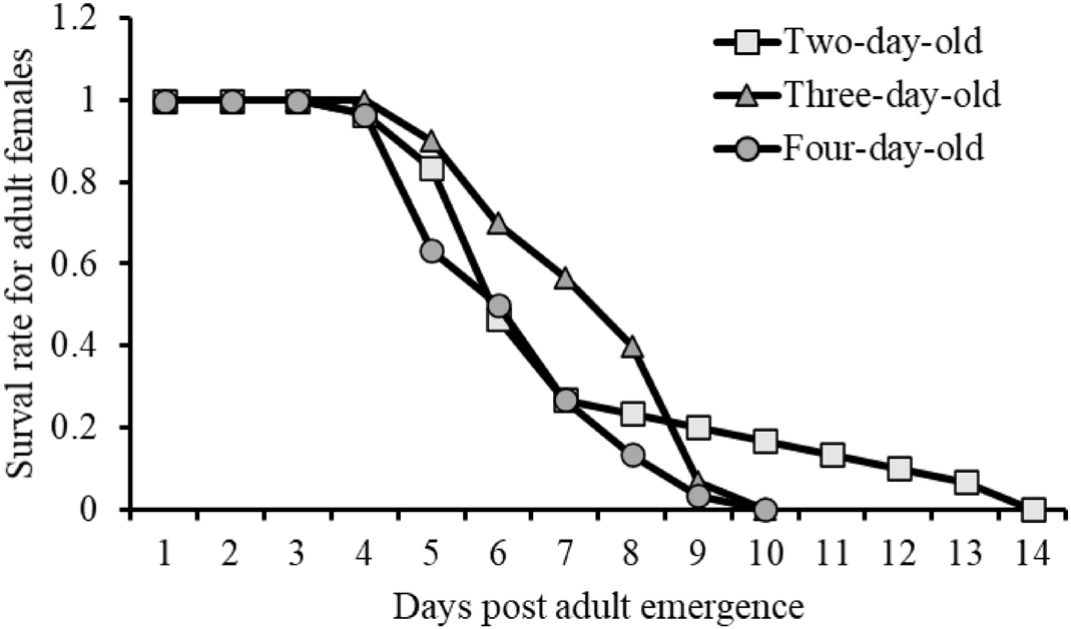
Survival rates for adult females of *T. javanus* exposed to two-, three-, and four-day-old *M. vitrata* caterpillars.

The host age at the time of oviposition did not significantly impact the mean longevity of adult females (GLM: χ2 = 0.81308, df = 2, *p* = 0.07). Adult females lived 5.46 ± 2.81 days (*n* = *30*), 5.63 ± 1.54 days (*n* = *30*) and 4.53 ± 1.569 days (*n* = *30*) when exposed to hosts that were two-, three-, and four-days-old, respectively. Survival of parasitoid females exposed to four-day-old hosts declined sharply after four days and reached zero after nine days, with that of three-day-old host displaying a similar pattern shifted by one day. While parasitoid females exposed to two-day-old hosts had a similar initial survival to those exposed to three-day-old host, their survival rate was the longest, reaching zero only after fourteen days (Fig. 6).

### Host age impact on offspring production

**Figure 7:**
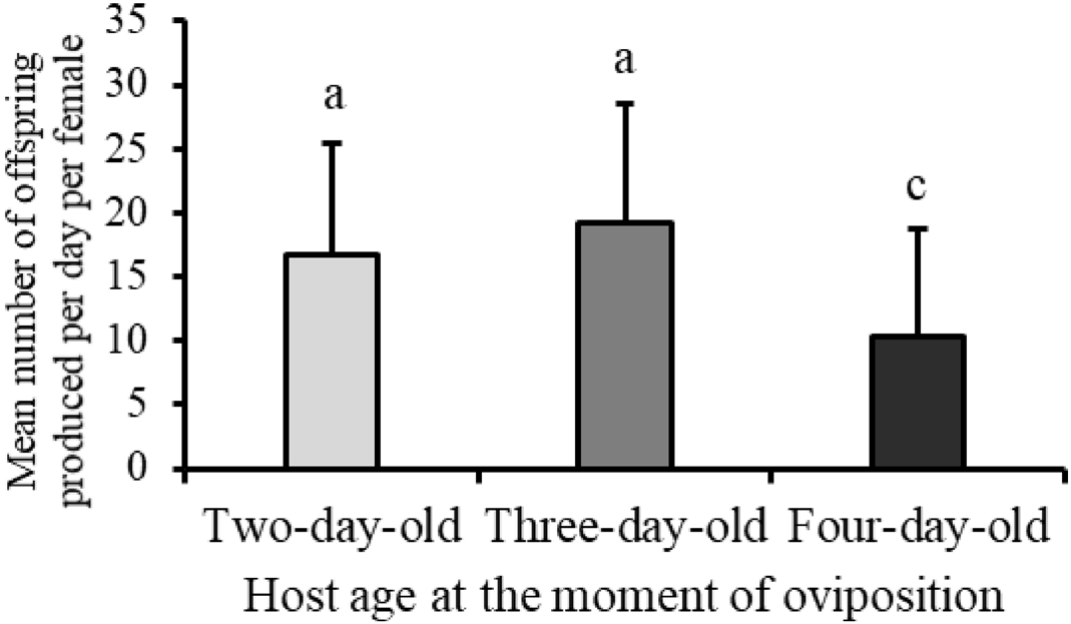
Mean offspring produced per female *T. javanus* exposed to two-, three-, and four-day-old *M. vitrata* caterpillars. Bars with the same letters indicate not significantly different (GLM and Tukey’s HSD test, *p* < 0.05).

The pre-oviposition period was similar whether females were exposed to two-, three-, or four-day-old hosts (*p* > 0.05) with 0.46 ± 0.32, 0.56 ± 0.12, and 0.3 ± 8.38 days, respectively. The host age at the time of oviposition significantly affected the mean number of offspring produced per female per day (GLM: χ2 = 350.27, df = 2, *p* < 0.001), with a higher mean number of offspring produced on two- and three-day-old hosts (Fig. 7).

**Figure 8:**
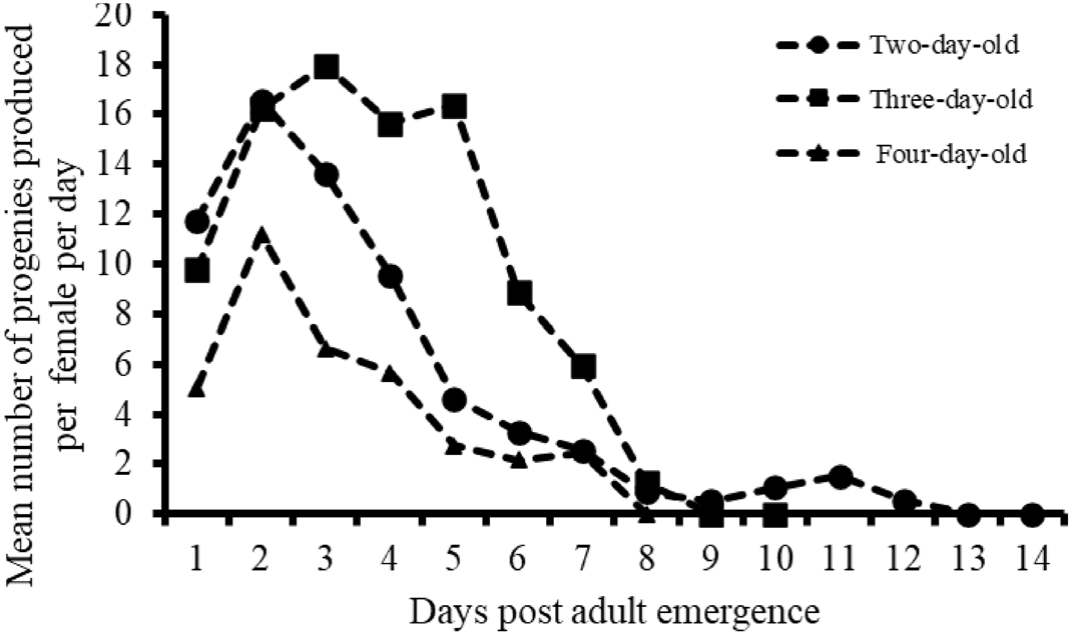
Dynamics of offspring produced daily per adult *T. javanus* female exposed to two-, three- and four-day-old *M. vitrata* caterpillars.

Females laid eggs until 12, 8, and 7 days post emergence, respectively, on two-, three-, or four-day-old *M. vitrata* caterpillars (Fig. 8). Mean number of offspring produced daily per female varied significantly (GLM: χ2 = 879.8, df = 26, *p* < 0.001), with the curves corresponding to offspring production per female showing that within the first seven days after emergence the mean number of offspring produced daily in four-day-old hosts was lower than on two- and three-day-old hosts (Fig. 8). Maximum average numbers of offspring produced daily per adult *T. javanus* female were 16.57 ± 2.09, 18.19 ± 2.36, and 11.2 ± 1.50, observed at day 2, 5, and 2 on two-, three- and four-day-old hosts, respectively (Fig. 8).

### Impact of host age on offspring sex-ratio

The sex-ratio was affected by the host age at the time of oviposition (χ2 = 261.09, df = 2, *p* < 0.001), and was male-biased on two- (χ2 = 197.92, df = 1, *p* < 0.001) and three- (χ2 = 739.6, df = 1, *p* < 0.001) but not four-day-old hosts (χ2 = 0.53432, df = 1, *p* > 0.05). Females exposed to three-day-old hosts produced the lowest proportion of females (0.24) compared to females exposed to two- (0.34) (χ2 = 58.216, df = 1, *p* < 0.001) and four-day-old hosts (0.51) (χ2 = 259.63, df = 1, *p* < 0.001). Similarly, females exposed to two-day-old hosts produced a lower proportion of females compared to females exposed to four-day-old hosts (χ2 = 81.996, df = 1, *p* < 0.001).

### Impact of host age on the life table parameters

The life table parameters of the parasitoid were significantly affected by the host age at the time of oviposition (Table 2). Higher average values of the intrinsic rate of increase (*r*) were recorded on two- (*p* < 0.01) and three-day-old hosts (*p* < 0.001) compared to four-day-old hosts. Similarly, higher average values of the rate of increase (*λ*), the net reproductive rate (*Ro*), and shorter doubling time (*TD*) were recorded on two- and three-day-old hosts compared to four-day-old hosts (*p* < 0.001).

**Table 2:** Life table parameters of *T. javanus* reared on two-, three- and four-day-old *M. vitrata* caterpillars (mean ± SE).

Host age	Parameters			
	*r* (day^-1^)	*λ*(day^-1^)	*Ro* (offspring)	*DT* (day)
Two-day-old	0.24 ± 0.01a	1.28 ± 0.01a	66.63 ± 6.62b	2.79 ± 0.07b
Three-day-old	0.26 ± 0.01a	1.29 ± 0.01a	92.03 ± 9.87a	2.66 ± 0.06b
Four-day-old	0.21 ± 0.01b	1.23 ± 0.01b	35.93 ± 6.51c	3.26 ± 0.16a

Means ± SE followed by different letters in the same column are significantly different among treatments using the paired bootstrap test at 5% significance level.

## Discussion

This study investigated the host stage preference of *T. javanus* adult females, it characterized the endoparasitic and ectoparasitic feeding behavior of the parasitoid larva, and evaluated the effect of host age on life history parameters of the parasitoid. *Therophilus javanus* preferred to oviposit in the first three larval instars of the host. Host larval instar and host age at the time of oviposition influenced offspring production and sex allocation. The higher intrinsic rate of increase (*r*) and the net reproductive rate (*Ro*) were recorded on three-day-old hosts, suggesting that the parasitoid population would increase more rapidly when provided three-day-old hosts. Inversely, the same three-day-old hosts produced the lowest proportion of daughter offspring.

In a choice situation, *T. javanus* preferred to oviposit in the first three larval instars of *M. vitrata*. In most cases, the two older instars resisted physically to the parasitoid and started to spin quickly a transparent silken web as a barrier to parasitoid attack (D. Aboubakar Souna, personal observation).

Life history observations of *T. javanus* have revealed three distinct larval stages, similar to the number of larval instars reported in other Braconidae wasps such as *Cardiochiles nigriceps* Viereck (Hymenoptera: Braconidae) and *Toxoneuron nigriceps* (Viereck) (Hymenoptera: Braconidae), larval endoparasitoids of *Heliothis virescens* (Fabricius) (Lepidoptera: Noctuidae)^22,23^. The first two larval stages of *T. javanus* feed inside the parasitized host larva, which continues to nourish itself and grow, while the third larval instar egresses from the host and continues host-feeding on it through the hole from which it egressed. Because parasitized *M. vitrata* caterpillars continue to feed and grow, we hypothesize that during the first two larval stages, *T. javanus* may have adapted to reduce the damage induced to vital organs of its host, such as feeding on the hemolymph and/or fat body. Unexpectedly, the third larval instar of *T. javanus* larva egressed destructively from the host body and continued to feed on it. This feeding behavior is similar to the one displayed by “tissue feeder” parasitoids^14^, and is utilized by some parasitoids to exploit a wide range of host stages^14^. However, after egression, some of them need to feed externally on the host tissue to acquire additional nutrients for successfully completing cocoon and adult stages^11^. As an example, *T. nigriceps* feeds on *H virescens* as a “tissue feeder” to regulate the host physiology, successfully completing its larval development after host egression^11^. Subsequent studies^10^ have confirmed that *T. nigriceps* exploits the dual larval feeding behavior to increase food acquisition and enhance its adult fitness. *T. javanus* may also use a similar adaptation strategy to regulate the host physiology for successfully completing its development. While under current rearing conditions the total larval duration of *M. vitrata* takes on average ten days^24^, the total development time of the larva until parasitoid egression was up to fourteen days, depending on the age of the larva at parasitization. This suggests that the developing parasitoids larva can actively manipulate the host metabolism to slow down its development and allow successful completion until egression.

To maximize the chance for oviposition in a suitable host, several parasitoids have developed host discrimination strategies for selecting suitable host stages that can increase their population fitness (c.f., ’mother knows best’^25^). On the other hand, some endoparasitoids can ensure the development of their progeny through maternal factors such as the venom fluid injected in the host at the time of parasitism^26^. Previous work has highlighted the presence of a venom gland attached to the reproductive tract of *T. javanus* females^4^ and hypothesized that the venom fluid might be secreted in the host during parasitism^27^. Although the venom function has not been elucidated in more detail to date, we speculate it could regulate metabolic processes in parasitized *M. vitrata* caterpillars in order to affect its development and hence ensure complete development of the parasitoid larva^28^.

The intrinsic rate of increase (*r*) obtained from the estimation of the life-table parameters indicate that the population of *T. javanus* would increase faster if reared on two and three-day-old *M. vitrata* caterpillars. However, three-day-old *M. vitrata* caterpillars induced the highest net reproductive rate (*Ro*), suggesting that oviposition by *T. javanus* adult females and subsequent feeding by developing immature stages on this host stage may engender a higher production of offspring and hence contribute positively to the overall population growth. Our results support previous observations reporting a higher suitability of two and three-day-old *M. vitrata* caterpillar to *Apanteles taragamae* (Viereck) (Hymenoptera: Braconidae)^24^. In the same vein, the second instar of *Spodoptera exigua* (Hübner) (Lepidoptera: Noctuidae) was identified as the most suitable host stage for *Microplitis similis* (Lyle) (Hymenoptera: Braconidae), based on the high values of *r* and *Ro*^29^. It was shown that parasitizing early host stages can reduce the development time in koinobionts parasitoids^16^; however, adult fitness of the offspring may be affected, in terms of short life expectancy, small size eggs, and reduced oviposition period^30^. Moreover, it has already been assessed that the development in early *M. vitrata* larval instars reduced the offspring potential fecundity in *T. javanus*^4^. Our observations further show that the sex-ratio of the offspring population was male-biased when stemming from three-day-old hosts, while the parasitoid produced more females when the oviposition occurred in four-day-old *M. vitrata* hosts. Similar occurrences have been reported in other parasitoids, e.g., the sex-ratio of *Diadegma mollipla* (Holmgren) (Hymenoptera: Ichneumonidae) was male-biased when the parasitoid developed in the early instars (L1) compared to the older instars (L2, L3 and L4) for the Diamondback Moth *Plutella xylostella* (Linnaeus) (Lepidoptera: Plutellidae)^31^. Environmental and genetic factors, as well as maternal decision can regulate sex allocation in Braconid parasitoids^32^. The genetic basis has been studied for species such as *Cotesia glomerata*, *Asobara tabida* and *Alysia manducator* (Hymenoptera: Braconidae)^33,34^. A more direct involvement of female parasitoids in sex allocation was proposed for *Diachasmimorpha longicaudata* (Ashmead) (Hymenoptera: Braconidae), observed to exploit physical stimuli and, perhaps more importantly, volatiles emitted by larvae of *Anastrepha fraterculus* (Wiedemann) (Diptera: Tephritidae)^35^. Similarly, during the oviposition process, female *T. javanus* may be able to use the same approach for discriminating host stages providing the best resources, maximizing chances for optimal progeny development and production of daughter offspring. However, the precise mechanisms for sex allocation in *T. javanus* will need to be elucidated by further investigations.

## Conclusions

Overall, the results provide key insights into the biology of *T. javanus* that can be useful to guide field releases of this parasitoid to control *M. vitrata* in West Africa. For optimizing the mass rearing of *T. javanus* in the laboratory, using three-day-old *M. vitrata* caterpillars (corresponding to first instar larvae) could accelerate the rate of colony production because of its higher net reproductive rate (*Ro*). However, knowing that female offspring was favored in older instars of *M. vitrata* caterpillar hosts, we suggest that a mix of first, second, and third instar larvae would be the best option for maximizing the total output of the mass rearing. Additional investigations are still required to assess the foraging behavior of the parasitoid in order to determine how females will detect and discriminate the most suitable stage or stages of *M. vitrata* caterpillars in the field.

## Material and methods

### Insect rearing

*Maruca vitrata* caterpillars were collected from the existing colony at International Institute of Tropical Agriculture (IITA) Benin laboratories, while the *T. javanus* colony was issued from individuals received from The World Vegetable Center (WorldVeg), Taiwan, Republic of China. Insect colonies were reared under laboratory conditions at the IITA-Benin research station following the methods described in^4^. Briefly, *M. vitrata* caterpillars were reared in large cylindrical plastic boxes (11 cm height × 16.5 cm diameter) containing sprouting cowpea grains. Colonies of *T. javanus* were maintained on *M. vitrata* first instar (three-day-old) caterpillars under confined conditions. For parasitism, *M. vitrata* caterpillars were submitted to three-day-old *T. javanus* adult mated females. Parasitized caterpillars were then reared until pupal stage on sprouting cowpea grains. Following emergence, *T. javanus* adults were fed on honey solution. Insects were maintained under laboratory conditions at 26 ± 1.1 °C, 76 ± 7% with 12: 12 L: D photoperiod.

### Influence of host stage on mother preference and progeny sex ratio

Five *M. vitrata* caterpillars (one each for larval instar 1 to 5) were exposed in a petri dish (90 mm in diameter × 15 mm in depth) to two-days-old female *T. javanus*, mated and with no preview oviposition experience. Before the experiment, the female parasitoids were provided with a drop of honey. The behavior of the parasitoid was observed during one hour and behavioral data were collected using Pocket Observer software (version 3.2; Noldus, The Netherlands). Females which did not make any oviposition attempt during the observation period were considered as non-responding and removed from the analysis. After each observation, larvae, female parasitoid, and the petri dish were replaced by the new ones. At the end of the observation period, each stung caterpillar was separated and placed individually in the small cup (3 cm diameter × 3.5 cm height), and offered fresh sprouting cowpea grains as feeding substrate until the formation of parasitoid cocoon. The collected cocoons were kept individually in the small cup (3 cm diameter × 3.5 cm height) until adult emergence and the sex ratio was noted in progeny population per host stage. A total of 72 responding females were considered for this observation.

### Larval feeding behavior and developmental time of immature stages

At first, three-day-old *M. vitrata* caterpillars were individually submitted to parasitism by three-day-old *T. javanus* mated females. Only a single stinging was allowed per caterpillar to ensure that a single parasitoid egg has been deposited. A total of forty-five (45) parasitized *M. vitrata* caterpillars were dissected under a macro zoom microscope (Olympus MVX10) at 24 h, 36 h and 48 h after parasitism, then daily until pre-pupae formation. *Therophilus javanus* immature stages present in the host body were removed and their image taken using an Olympus XC50 camera. The shape of larval body and mandibles were used to distinguish the three larval instars of *T. javanus*. The development of forty-one (41) pupae was observed until adult emergence.

### Impact of host age on adult parasitoid development time

To determine the impact of the host age on *T. javanus* development time from egg to adult, a total of one hundred sixty (160) two-, three- or four-day-old *M. vitrata* caterpillars were individually submitted to parasitism. One-time stinging was allowed per caterpillar. Parasitized caterpillars were reared until cocoon formation and adult emergence as previously described. Each cocoon was separated in individual small cup (3 cm diameter × 3.5 cm height) containing a honey drop. Cups were observed daily until adult emergence.

### Impact of host age on the life table parameters

Thirty (30) newly emerged *T. javanus* couples were transferred to a rearing unit consisting of a plastic cup (diameter: 9 cm at the base and 12 cm at the top; height: 4.5 cm) containing sprouting cowpea grains and thirty (30) two-, three- or four-day-old *M. vitrata* caterpillars, respectively. Because the mating behavior had not been studied in this parasitoid, couples were allowed to mate all the time during the experiment. Each couple was allowed to parasitize a given host age during 24 h, and this, every day until female death. If the male died, it was replaced by another male. After exposure to parasitism, *M. vitrata* caterpillars remained in the rearing unit feeding on sprouting cowpea grains until parasitoid cocoon formation. For each couple and each day of exposure, the cocoons formed were regularly collected and placed in another plastic cup containing a drop of honey. Offspring emergence was recorded daily, and the sex of each progeny was noted. Life table parameters were estimated as described in^36^: the adult female survival rate, the finite rate of increase (λ), the intrinsic rate of increase (*r*), the net reproductive rate (*Ro*), and doubling time (*DT*).

### Statistical analysis

The Shapiro–Wilk normality test was realized to test the normal distribution of data^37^. A general linear model (GLM) with a gamma distribution was used to investigate the effect of host age at the time of oviposition on the longevity of mother parasitoids and the development time of offspring. GLM with Poisson errors and log-link functions, corrected for over-dispersion, were used to investigate the influence of host age on the mean offspring produced per day, and the mean offspring produced daily per female. General linear models (GLM) with binomial errors distribution and logit-link function corrected for over-dispersion were used to analyze the proportion of oviposition per host larval instars. Means were compared using the ‘glht’ function in the “multcomp” package of the R software^38^. The impact of host larval instar and host age at the time of oviposition on offspring sex allocation was tested by a chi-square test followed by a post-hoc test using the ‘pairwise nominal independence’ function in the “rcompanion” package of the R software^39^. All these tests were conducted with R software package R 3.5.3^40^. The life table parameters were calculated following standard methodology^41,42^ using the TWOSEX-MSChart software^43^. The impact of host age at the time of oviposition on the duration of the oviposition periods and each life table parameter was accessed using the paired bootstrap test at *P* = 5%^36^. To generate stable estimates of standard errors of the duration of the oviposition periods and each life table parameter we used the bootstrap value of 100,000^44^.

## Data Availability

The datasets generated during and/or analyzed during the current study are available from the corresponding author on reasonable request.
